# Effects of long-term care insurance on women’s time allocation: evidence from pilot regions in China

**DOI:** 10.3389/fpubh.2026.1841498

**Published:** 2026-07-20

**Authors:** Lin Wei, Ying Liu, Linping Wang

**Affiliations:** College of Economics and Management, Fujian Agriculture and Forestry University, Fuzhou, China

**Keywords:** long-term care insurance, market labor, leisure, household labor, women’s welfare

## Abstract

**Introduction:**

As population aging intensifies in China, the demand for long-term care continues to rise, posing challenges to the sustainability of the traditional family-based care model. As important providers of family care, women often face a trade-off between caregiving responsibilities and personal development. In this context, long-term care insurance (LTCI) may support family caregiving and affect women’s time allocation.

**Methods:**

Using data from the 2016 and 2018 waves of the China Family Panel Studies (CFPS), this study examines changes in women’s time allocation associated with LTCI pilot policy exposure. The final analytical sample includes 11,475 female observations, with 1,426 in the treatment group and 10,049 in the control group. The study combines propensity score matching with a before-and-after comparison between pilot and non-pilot regions.

**Results:**

The study finds that LTCI pilot policy exposure is significantly associated with increased market labor time and leisure time and reduced household labor time among women. These associations vary across regions, age groups, and income groups. The mechanism analysis suggests that caregiving burden and upward financial support may serve as potential channels. Further analysis suggests that LTCI pilot policy exposure may be associated with improved intergenerational emotional bonds through increased leisure time.

**Discussion:**

This study provides empirical evidence for improving the LTCI system and enhancing women’s well-being.

## 1 Introduction

Improvements in medical care and declining fertility have accelerated global population aging. As the country with the world’s largest older adult population, China faces particularly severe aging challenges. According to the 2025 Statistical Bulletin on the Development of China’s Aging Undertakings (1), by the end of 2024, China’s population aged 60 and above had reached approximately 321 million, accounting for 22.8% of the total population. This population group is projected to peak at 487 million around 2053. Due to aging and disease, the risk of disability among older adults has increased substantially. Currently, the number of disabled and semi-disabled older adults in China has reached 44 million. By 2050, the total number of moderately and severely disabled individuals aged 65 and above is expected to reach 83 million (2). The growing number of disabled older adults has led to a sustained increase in the demand for long-term care services.

Despite the growing demand for formal long-term care services, families remain the primary source of care for disabled older adults, with women shouldering the majority of caregiving responsibilities. The 2021 Survey on the Living Conditions of the Older Adults in China shows that 93.1% of long-term care is provided by family members (3). Based on the Third Survey on the Social Status of Women in China, 43.1% of women undertake primary caregiving roles, significantly higher than the 19.4% of men (4). This indicates a pronounced gender disparity in family caregiving.

This gendered division of labor may crowd out women’s time for personal development. Given limited time resources, increased caregiving responsibilities force women to reallocate their time. Accordingly, this study treats family caregiving as a constraint and examines its impact on three main categories of women’s time allocation: market labor, leisure, and household labor (excluding caregiving). In terms of market labor, intensive caregiving reduces women’s market labor supply. Chen et al. (5) find that employed female caregivers reduce their weekly market labor time by 2.8–4.8 h due to caregiving responsibilities. Compared with men, they are also more likely to retire early (6). In terms of leisure, caregiving similarly encroaches on women’s free time. Studies show that caregiving responsibilities significantly reduce time spent on physical activities, hobbies, and social life, with women being more affected (7, 8). Married employed female caregivers lose on average about 5 h of leisure time per week (9), and their satisfaction with leisure also declines (10). In terms of household labor, caring for disabled older adults is often accompanied by additional unpaid household work, further increasing women’s burden. Overall, family caregiving reshapes women’s time allocation by reducing market labor supply and leisure time while increasing non-care household labor, thereby constraining their long-term development.

In response to the growing long-term care needs of an ageing population, China officially launched the first batch of Long-Term Care Insurance (LTCI) pilot programs in 2016. LTCI provides basic daily care and related medical services for eligible disabled older adults. It may also subsidize formal care costs through partial reimbursement, and in some regions further offers financial compensation to family caregivers. The implementation of this policy may affect women’s time allocation by reshaping the intra-household division of caregiving responsibilities.

Furthermore, whether LTCI affects emotional connections between women and their parents also warrants attention. The 2022 Report on Care Consumption Among Older Adults indicates that home-based care remains the predominant mode in China (88.93%), while institutional care accounts for only 11.07%, highlighting older adults’ heavy reliance on family care and emotional support (11). Although LTCI helps alleviate caregiving burdens, it may affect the time family members spend with disabled older adults, thereby influencing intergenerational emotional connections. The overall impact remains to be further examined.

In summary, based on data from the 2016 and 2018 waves of the China Family Panel Studies (CFPS), this study adopts propensity score matching and compares changes between pilot and non-pilot regions before and after policy implementation to examine women’s time allocation associated with LTCI pilot policy exposure. The main contributions of this study are threefold. First, from the perspective of women, it examines the association between LTCI pilot policy exposure and multidimensional time allocation, thereby extending the analytical framework for understanding the welfare implications of LTCI policy. Second, it uses propensity score matching to enhance comparability between groups, helping reduce selection bias from observable characteristics and improve the credibility of the estimates. Third, it extends the analysis to the family level by examining the association between LTCI pilot policy exposure and intergenerational emotional bonds, enriching the understanding of the broader implications of LTCI policy.

## 2 Literature review

Existing studies have mainly focused on the effects of LTCI on the health status and economic resources of disabled older adults. By improving disabled older adults’ ability to afford formal care services, LTCI increases the demand for formal care services (12, 13). Meanwhile, LTCI also has positive effects on the physical and mental health of disabled older adults (14, 15). At the household level, LTCI helps reduce medical expenditures and lowers the financial risk of falling into poverty due to illness (16, 17).

In terms of mechanisms, LTCI partially substitutes for family caregiving responsibilities by providing formal care services. Previous studies have shown that, after policy implementation, formal care services partly replace family care (13), and the proportion of adult children living with their parents declines significantly (18), thereby facilitating family caregivers’ labor market participation. Zhang et al. (19) further find that LTCI increases the non-agricultural employment rate of family caregivers.

In addition, existing studies have examined the effects of LTCI on intergenerational support, but mainly in terms of financial support and caregiving support. Relevant studies show that LTCI effectively reduces children’s upward financial support and caregiving burden (20, 21).

However, existing research has paid limited attention to the effects of LTCI on female family members. Existing studies have mostly focused on changes in market labor time, while giving relatively insufficient attention to other important dimensions of time use, such as leisure time and household labor time. Meanwhile, changes in women’s time allocation may further affect intergenerational emotional bonds, but this household-level policy effect has not been fully discussed. Therefore, it is necessary to systematically examine the effects of LTCI on women’s multidimensional time allocation and intergenerational emotional bonds from the perspective of women.

## 3 Theoretical analysis and hypothesis

In practice, under the combined influence of the gendered division of family roles and gender wage disparities, women often assume more caregiving responsibilities when their parents become disabled. Because time resources are limited, increased time devoted to caregiving may crowd out time for other activities. Against this background, long-term care insurance, as an institutional arrangement that provides care services and financial support for disabled older adults, may affect women’s time allocation. Based on Becker’s (22) time allocation theory, this study divides women’s time into three dimensions: market labor, leisure, and household labor, and analyzes the possible mechanisms through which long-term care insurance affects these dimensions.

Existing studies suggest that long-term care insurance may affect family caregivers’ time allocation through two mechanisms (23). The first is the substitution effect, whereby LTCI provides formal care to replace part of family care, which may reduce caregivers’ time constraints and increase the time available for other activities. The second is the income effect, whereby LTCI provides financial subsidies to family caregivers but may have limited effects on caregiving-related time constraints if no supplementary care services are provided.

Under the substitution effect mechanism, when LTCI lowers the cost of formal eldercare services relative to the opportunity cost of family care, families may replace part of informal care with formal care (24), thereby freeing up women’s available time. On the one hand, working-age women are often at a critical stage of career development, and labor participation tends to be a high priority under the influence of economic pressure, career advancement, and social recognition. Therefore, after the caregiving burden is reduced, the freed-up time may be reallocated to market labor to maintain or increase labor participation. At the same time, greater engagement in market labor may increase the opportunity cost of household labor, thereby reducing household labor time. On the other hand, the freed-up time may also be shifted to leisure. In labor economics, leisure is regarded as a consumption good relative to market labor. According to the work-leisure choice model, individuals need to trade off labor income against leisure; when income levels rise, the demand for leisure may increase. Therefore, after the caregiving burden is reduced, some women may allocate part of the freed-up time to leisure, thereby improving subjective well-being.

Under the income effect mechanism, LTCI may ease economic constraints by providing financial support or reducing expenditure pressure. Existing studies have shown that an increase in women’s relative income can enhance their household bargaining power and promote a more equitable division of household labor (25). Because routine household labor is highly substitutable, an increase in economic resources may reduce women’s involvement in such activities. In addition, according to Becker’s (26) altruistic utility theory, adult children may provide financial support to their disabled parents to ensure their quality of life and meet their medical care needs. However, related studies have shown that LTCI can effectively reduce the number of medical visits and medical expenses among disabled older adults (27), thereby reducing their economic dependence on adult children (28). This change may help improve the economic situation of adult daughters, further enhance their household bargaining power, and reduce their household labor burden to some extent. Based on the above analysis, this study proposes the following hypothesis:

*H1*: LTCI increases women’s market labor time and leisure time while reducing household labor time.

*H2a*: Reducing women’s caregiving burden is a key mechanism through which LTCI affects market labor time and leisure time.

*H2b*: Increasing women’s economic resources is a key mechanism through which LTCI affects household labor time.

In China’s traditional eldercare model, women not only provide daily living care but also play an important role in emotional support (29). For disabled older adults, in addition to basic care needs and medical support, emotional support from adult children is also an important source of support for maintaining their physical and mental health. However, long-term and intensive caregiving can increase women’s psychological stress (30), which may weaken their ability to provide emotional support.

Against this background, long-term care insurance may affect intergenerational emotional bonds between women and their parents by changing women’s time allocation. Specifically, the care substitution effect may free up some of the time women spend on caregiving, allowing them to reallocate this time between market work and leisure. Increased leisure time may create more opportunities for adult daughters to spend time with their parents, communicate with them, and express affection. Previous studies have shown that shared leisure activities facilitate family communication and intergenerational interaction (31, 32), whereas longer working hours may reduce the frequency with which adult children visit their older parents (33). Therefore, spending more of the freed-up time on leisure rather than market work may be more conducive to strengthening intergenerational emotional bonds. In addition, long-term care insurance may affect women’s household labor time through an income effect. However, how the time released from household labor is subsequently reallocated remains uncertain, and its implications for intergenerational emotional bonds are therefore unclear. Leisure time may thus represent a potential channel through which long-term care insurance affects intergenerational emotional bonds. Based on the above analysis, this study proposes the following hypothesis:

*H3*: LTCI strengthens women’s emotional bonds with their parents by increasing their leisure time.

## 4 Materials and methods

### 4.1 Identification strategy

In June 2016, China announced the first batch of LTCI pilot programs, covering 15 cities and 2 provinces^1^. Although some regions had previously conducted small-scale trials, these did not generate substantial institutional impact. Due to implementation lags, the LTCI system was fully operational in all pilot areas only by early 2018. Therefore, this study defines 2016 as the pre-policy period (post = 0) and 2018 as the post-policy period (post = 1); pilot areas are defined as the LTCI policy-exposure group (treat = 1), and non-pilot areas as the control group (treat = 0). On this basis, this study adopts a quasi-experimental research design and employs two models to examine the association between LTCI policy exposure and women’s time allocation. Model 1 estimates the association based on differences in outcomes between pilot and non-pilot areas before and after policy implementation. Model 2 further combines matching on observable characteristics with the above comparison strategy to improve sample comparability and the reliability of the estimates.

Specifically, this study first uses a logit model to estimate the probability of being in the LTCI policy-exposure group based on observable characteristics and calculates the propensity score:

$$p\;(X_{i})=\mathrm{Pr}\;(\text{Trea}t_{i}=1|X_{i})=F\;\;(\;h\;(\;X_{i})\;)$$

Where $X_{i}$ denotes individual characteristics, $p(X_{i})$ is the propensity score, and $\text{Trea}t_{i}$ indicates whether the individual belongs to the LTCI policy-exposure group. Based on the estimated propensity scores, kernel-based matching with a bandwidth of 0.01 is used to construct the matched sample.

The specific estimation equation is as follows:

$$Y_{\mathrm{it}}=\beta_{0}+\beta ₁\text{Trea}t_{i}+\beta ₂\mathrm{Pos}t_{t}+\beta ₃(\text{Trea}t_{i}\times \mathrm{Pos}t_{t})+\beta ₄X_{\mathrm{it}}+\varepsilon_{\mathrm{it}}$$

Where i denotes individuals, and t=0,1 corresponds to the periods before and after policy implementation. $Y_{\mathrm{it}}$ represents the outcome variable for individual i at time t ; ${\text{Treat}}_{i}$ indicates whether the individual belongs to the LTCI policy-exposure group; ${\text{Post}}_{t}$ denotes whether the observation occurs after policy implementation; the interaction term ${\text{Treat}}_{i}\times {\text{Post}}_{t}$ captures the average effect of LTCI policy exposure; $X_{\mathrm{it}}$ is a set of control variables; and $\varepsilon_{\mathrm{it}}$ represents the error term. The same specific estimation equation is used for both the heterogeneity analysis and the potential mechanism analysis, with the dependent variables replaced accordingly.

### 4.2 Data sources

This study uses data from the 2016 and 2018 waves of the China Family Panel Studies (CFPS), a nationally representative longitudinal household survey conducted by Peking University, covering population, education, employment, income, health, family relationships, and time use.

The sample is selected and processed as follows. (1) The sample is restricted to female respondents from the adult questionnaire. (2) The sample is restricted to individuals aged 16–60. (3) The sample is restricted to observations with at least one living parent, based on parental information matched via household identifiers. (4) Observations are excluded if any of household labor time, market labor time, or leisure time exceed 168 h per week. (5) Observations with missing values in key variables, including market labor time, gender, age, and place of residence, are excluded. (6) For policy identification, the sample is restricted to four pilot regions—Chongqing, Shanghai, Shandong, and Jilin—which constitute the treatment group; observations from provinces with partial pilot implementation are excluded^2^, and non-pilot provinces serve as the control group.

Chongqing, Shanghai, Shandong, and Jilin are selected as the treatment group for two reasons. First, due to data limitations, detailed city-level identifiers are unavailable. Second, since the introduction of the long-term care insurance policy in 2016, only these four provincial-level regions have implemented the policy across the entire jurisdiction, whereas Anhui, Sichuan, Hebei, Guangdong, Hubei, Jiangsu, Zhejiang, Jiangxi, Xinjiang, and Heilongjiang have implemented it only in selected cities. Therefore, including provinces with partial pilot implementation may lead to the misclassification of individuals not covered by the policy as treated, thereby introducing measurement error into the estimation of policy effects.

After these procedures, the final sample consists of 11,475 observations, including 1,426 in the treatment group and 10,049 in the control group^3^.

### 4.3 Selection of variables

#### 4.3.1 Dependent variables

Following relevant studies (34), this study uses market labor time, leisure time, and household labor time as dependent variables for women’s time allocation. At the same time, intergenerational closeness, frequency of face-to-face meetings, and frequency of phone calls are selected as measures of intergenerational emotional ties.

#### 4.3.2 Core explanatory variables

The core explanatory variable is the implementation of LTCI. According to the first-round pilot policy, samples whose parents are located in Shanghai, Chongqing, Shandong, or Jilin in 2018 are assigned a value of 1, while all other samples are assigned 0. Although actual LTCI participation or benefit receipt cannot be precisely identified, parents living in pilot provinces are treated as potentially exposed to the policy. Thus, the treatment variable captures regional policy exposure rather than actual LTCI benefit receipt, and the estimates should be interpreted as policy exposure effects.

#### 4.3.3 Potential mechanism variables

This study uses Care and Financial support to examine the potential mechanisms linking LTCI to women’s time allocation. Due to the lack of information on the utilization of formal care services and detailed caregiving hours in the CFPS, Care is used as a proxy variable for caregiving burden. Meanwhile, given the absence of direct data on LTCI cash subsidies in the CFPS, Financial support is employed as a proxy variable for intergenerational financial support provided to parents.

#### 4.3.4 Control variables

Following relevant studies (24, 35), this study includes covariates at both individual and household levels. Individual covariates include age, residence, marital status, education, self-rated health, self-employment status, and relative income. Household covariates include father’s chronic illness, mother’s chronic illness, number of children, and number of living parents. Detailed variable definitions and coding schemes are presented in Table 1.

**Table 1:** Variable definitions.

Variables type	Variables name	Definition	Min	Max
Dependent variables	Market labor time	Weekly market labor hours	0	168
	Leisure time	Sum of weekly hours spent watching movies, exercising, and browsing the internet in spare time	0	155.5
	Household labor time	Weekly household labor hours	0	112
	Intergenerational closeness	Very close = 5; Close = 4; Average = 3; Not close = 2; Not close at all = 1	1	5
	Frequency of face-to-face meetings	Daily = 6; 3–4 times/week = 5; 1–2 times/week = 4; 2–3 times/month = 3; Monthly = 2; Every few months = 1; Never = 0	0	6
	Frequency of phone calls	Daily = 6; 3–4 times/week = 5; 1–2 times/week = 4; 2–3 times/month = 3; Monthly = 2; Every few months = 1; Never = 0	0	6
Core explanatory variables	LTCI	Either parent in SH, CQ, SD, or JL in 2018 = 1; Otherwise = 0	0	1
Mediating factors	Care	The respondent is the primary caregiver when their parents are ill = 1; otherwise = 0	0	1
	Financial support	Financial support provided to parents	0	50,000
Control variables	Age	Age of women	16	60
	Residence	Urban = 1; Rural = 0	0	1
	Marital Status	Married = 1; Others (unmarried, cohabiting, divorced, widowed) = 0	0	1
	Education	PhD = 21; Master = 18; Bachelor = 16; Associate = 15; High school = 12; Middle school = 9; Primary school = 6; Below primary school/Illiterate = 0	0	21
	Self-rated health	Very healthy = 5; Healthy = 4; Relatively healthy = 3; Fair = 2; Unhealthy = 1	1	5
	Relative income	Very high = 5; High = 4; Average = 3; Low = 2; Very low = 1	1	5
	Self-employment status	Self-employed = 1, Employed = 0	0	1
	Father’s chronic illness	Yes = 1; no = 0	0	1
	Mother’s chronic illness	Yes = 1; no = 0	0	1
	Number of children	Number of children	0	9
	Number of living parents	Number of living parents	1	2

## 5 Results

### 5.1 Descriptive statistics

Table 2 presents descriptive statistics. Overall, women’s weekly market labor time averages approximately 31.28 h, leisure time about 20.17 h, and household labor time about 15.35 h. In terms of time allocation, market labor time accounts for the largest share, followed by leisure time and household labor time.

**Table 2:** Descriptive statistics.

Variables type	Variables	Full		Treated		Control	
		M	SD	M	SD	M	SD
Dependent variables	Market labor time	31.284	26.770	30.373	25.687	31.414	26.919
	Leisure time	20.172	18.272	22.344	17.828	19.864	18.314
	Household labor time	15.353	12.496	13.959	11.463	15.551	12.624
	Intergenerational closeness	3.275	1.945	3.456	1.922	3.249	1.947
	Frequency of face-to-face meetings	2.483	2.180	2.922	2.296	2.421	2.156
	Frequency of phone calls	2.570	2.202	2.726	2.270	2.548	2.191
Mediating factors	Care	0.261	0.409	0.241	0.402	0.265	0.410
	Financial support	115.147	586.642	160.826	494.310	108.665	598.331
Control variables	Age	36.661	11.911	38.116	12.027	36.454	11.881
	Residence	0.500	0.500	0.676	0.468	0.475	0.499
	Marital Status	0.787	0.409	0.767	0.423	0.790	0.407
	Education	7.829	5.148	9.400	5.132	7.606	5.112
	Self-rated health	3.097	1.178	3.072	1.172	3.101	1.179
	Relative income	2.039	1.267	2.204	1.212	2.015	1.273
	Self-employment status	0.339	0.473	0.224	0.417	0.355	0.479
	Father’s chronic illness	0.173	0.210	0.175	0.224	0.172	0.208
	Mother’s chronic illness	0.233	0.244	0.241	0.270	0.232	0.240
	Number of children	1.246	1.030	0.918	0.720	1.292	1.058
	Number of living parents	1.776	0.417	1.764	0.425	1.778	0.416

To reduce the impact of extreme values on the regression results, all continuous time variables in this paper are increased by 1 and then log-transformed.

A further comparison between the treatment and control groups shows that women in the treatment group have an average market labor time of 30.37 h, slightly lower than 31.41 h in the control group. This difference may be related to sample characteristics: women in the treatment group are older on average, and their parents have a higher prevalence of chronic diseases, which increases caregiving burdens and limits participation in market labor. At the same time, women in the treatment group report higher average leisure time and lower average household labor time than the control group, a pattern that may partly reflect the effects of LTCI implementation. Therefore, it is necessary to further control for between-group differences in the subsequent regression analysis.

### 5.2 Balance test after propensity score matching

To enhance the comparability between the treatment and control groups, this study employs kernel-based propensity score matching with a bandwidth of 0.01 and performs a covariate balance test on the matched sample. Table 3 shows that, after matching, the absolute values of standardized deviations for all covariates are below 10%, and the standardized deviations have significantly decreased, indicating that between-group comparability has been effectively improved. After excluding unmatched samples, a total of 11,425 valid observations are obtained.

**Table 3:** The results of the balance test.

Variables	Unmatch	Mean		% Reduct		t-test	
	Matched	Treated	Control	% bias	Bias	*t*	*p* > *t*
Age	U	38.116	36.454	13.9		4.94	0.000
	M	38.101	37.609	4.1	70.4	1.08	0.281
Residence	U	0.676	0.475	41.5		14.33	0.000
	M	0.676	0.678	−0.5	98.8	−0.13	0.895
Marital status	U	0.767	0.790	−5.5		−1.96	0.051
	M	0.768	0.762	1.4	74.7	0.36	0.718
Education	U	9.400	7.606	35.0		12.40	0.000
	M	9.400	9.477	−1.5	95.7	−0.41	0.684
Self-rated health	U	3.072	3.101	−2.5		−0.87	0.383
	M	3.072	3.100	−2.5	0.9	−0.67	0.505
Relative income	U	2.204	2.015	15.2		5.28	0.000
	M	2.202	2.201	0.1	99.4	0.02	0.981
Self-employment status	U	0.224	0.355	−29.2		−9.83	0.000
	M	0.224	0.220	0.8	97.1	0.24	0.807
Father’s chronic illness	U	0.175	0.172	1.0		0.37	0.714
	M	0.175	0.173	0.8	20.5	0.21	0.836
Mother’s chronic illness	U	0.241	0.232	3.8		1.41	0.158
	M	0.241	0.239	0.8	78.8	0.21	0.837
Number of children	U	0.918	1.292	−41.3		−12.93	0.000
	M	0.919	0.892	3.0	92.8	0.95	0.344
Number of living parents	U	1.764	1.778	−3.2		−1.14	0.256
	M	1.765	1.769	−0.9	71.7	−0.24	0.811

### 5.3 Baseline regression

Table 4 reports the baseline associations between LTCI policy exposure and women’s time allocation. The estimation results of Model 1 and Model 2 are generally consistent, suggesting that the findings are relatively robust. Columns (1) and (2) show that LTCI policy exposure is significantly associated with increases in women’s market labor time by 27.4 and 15.6%, respectively. Columns (3) and (4) show that LTCI policy exposure is significantly associated with increases in women’s leisure time by 9.0 and 14.2%, respectively. Columns (5) and (6) indicate that LTCI policy exposure is significantly associated with reductions in women’s household labor time by 22.3 and 16.4%, respectively. Overall, the empirical results support Hypothesis 1, suggesting that LTCI policy exposure is associated with changes in women’s time allocation patterns.

**Table 4:** Regression results.

Variable	Market labor time		Leisure time		Household labor time	
	Model 1	Model 2	Model 1	Model 2	Model 1	Model 2
	(1)	(2)	(3)	(4)	(5)	(6)
LTCI	0.242*** (0.083)	0.145* (0.087)	0.086* (0.048)	0.133*** (0.048)	−0.252*** (0.056)	−0.179*** (0.055)
Age	−0.035*** (0.002)	−0.051*** (0.003)	−0.005*** (0.001)	−0.003* (0.002)	0.013*** (0.001)	0.018*** (0.002)
Residence	−0.074** (0.035)	−0.048 (0.057)	0.258*** (0.023)	0.255*** (0.034)	0.049** (0.020)	0.023 (0.030)
Marital Status	−0.580*** (0.045)	−0.438*** (0.066)	−0.077** (0.030)	−0.113*** (0.042)	0.294*** (0.028)	0.266*** (0.041)
Education	0.011* (0.004)	0.009 (0.006)	0.045*** (0.003)	0.032*** (0.004)	−0.004* (0.002)	−0.008** (0.003)
Self-rated health	−0.013 (0.015)	−0.032 (0.023)	−0.013 (0.010)	−0.007 (0.015)	−0.010 (0.009)	0.017 (0.013)
Relative income	0.216*** (0.013)	0.248*** (0.020)	0.225*** (0.010)	0.173*** (0.014)	0.206*** (0.009)	0.144*** (0.013)
Self-employment status	0.455*** (0.039)	0.391*** (0.066)	0.036 (0.024)	−0.039 (0.039)	0.264*** (0.021)	0.140*** (0.032)
Father’s chronic illness	0.208*** (0.070)	0.332*** (0.088)	−0.121** (0.047)	−0.038 (0.060)	−0.046 (0.044)	−0.046 (0.063)
Mother’s chronic illness	0.069 (0.061)	0.134* (0.081)	−0.013 (0.040)	0.001 (0.049)	0.064* (0.038)	0.054 (0.054)
Number of children	0.122*** (0.022)	0.182*** (0.044)	−0.090*** (0.014)	−0.097*** (0.026)	0.051*** (0.013)	0.095*** (0.023)
Number of living parents	0.073* (0.042)	0.083 (0.064)	−0.061** (0.026)	−0.063* (0.038)	−0.024 (0.022)	−0.015 (0.033)
_cons	3.378*** (0.135)	3.784*** (0.213)	2.136*** (0.087)	2.337*** (0.126)	1.181*** (0.075)	1.110*** (0.116)
*R*-squared	0.101	0.158	0.163	0.129	0.206	0.193
Sample size	11,475	11,416	11,475	11,416	11,475	11,416

*p < 0.1, ** *p* < 0.05, and *** *p* < 0.01. Standard errors clustered at the individual level are reported in parentheses.

### 5.4 Robustness checks

#### 5.4.1 Alternative bandwidths

To examine whether the results are sensitive to the choice of matching bandwidth, this study conducts robustness checks by varying the kernel matching bandwidth from 0.02 to 0.1. The estimates obtained under different bandwidth settings are generally consistent with the baseline results, suggesting that the main findings are not driven by a specific bandwidth choice. Due to space constraints, Table 5 reports only the results using a bandwidth of 0.08. These results provide additional support for the stability of the estimated associations between LTCI policy exposure and women’s time allocation.

**Table 5:** Alternative bandwidths.

Variable	Market labor time	Leisure time	Household labor time
	(1)	(2)	(3)
LTCI	0.157* (0.087)	0.130*** (0.048)	−0.185*** (0.055)
*R*-squared	0.152	0.135	0.195
Control variables	Controlled	Controlled	Controlled
Sample size	11,474	11,474	11,474

*p < 0.1 and *** *p* < 0.01. Standard errors clustered at the individual level are reported in parentheses.

#### 5.4.2 Alternative matching method

To examine whether the results are sensitive to the choice of matching method, this study replaces the kernel matching used in the baseline regression with nearest-neighbor matching combined with a caliper. Specifically, 1:4 nearest-neighbor matching is adopted, with the caliper set at 0.01. The results are shown in Table 6. Compared with kernel matching, nearest-neighbor matching with a caliper imposes stricter restrictions on the matching distance, resulting in the exclusion of 6,301 unmatched observations. After changing the matching method, the estimated associations between LTCI policy exposure and women’s time allocation remain generally consistent with the baseline results, suggesting that the main findings are not sensitive to a specific matching method.

**Table 6:** Alternative matching method.

Variable	Market labor time	Leisure time	Household labor time
	(1)	(2)	(3)
LTCI	0.169** (0.086)	0.113** (0.049)	−0.198*** (0.056)
*R*-squared	0.146	0.138	0.205
Control variables	Controlled	Controlled	Controlled
Sample size	5,174	5,174	5,174

**p < 0.05 and ***p < 0.01. Standard errors clustered at the individual level are reported in parentheses.

#### 5.4.3 Controlling for the propensity score

To examine the robustness of the Model 2 results, this study follows Zhang et al. (36) by incorporating the propensity score of being in the LTCI policy-exposure group as a control variable into Model 1, without conducting sample matching. The results shown in Table 7 indicate that the estimates obtained after controlling for the propensity score are generally consistent with the baseline results, and some coefficients are closer to the matched estimates. This provides additional support for the robustness of the estimated associations between LTCI policy exposure and women’s time allocation.

**Table 7:** Controlling for the propensity score.

Variable	Market labor time	Leisure time	Household labor time
	(1)	(2)	(3)
LTCI	0.252*** (0.083)	0.087* (0.048)	−0.250*** (0.056)
*R*-squared	0.103	0.163	0.206
Control variables	Controlled	Controlled	Controlled
Sample size	11,475	11,475	11,475

*p < 0.1 and ***p < 0.01. Standard errors clustered at the individual level are reported in parentheses.

#### 5.4.4 Winsorization of dependent variables

To reduce the influence of extreme values, market labor time, leisure time, and household labor time are winsorized at the 1% level and then log-transformed before re-estimating the regressions. The results are reported in Table 8. In both Model 1 and Model 2, the signs and significance levels of the key coefficients remain generally consistent with the baseline results, suggesting that the estimated associations are not sensitive to the treatment of extreme values.

**Table 8:** Winsorization of dependent variables.

Variable	Market labor time		Leisure time		Household labor time	
	Model 1	Model 2	Model 1	Model 2	Model 1	Model 2
	(1)	(2)	(3)	(4)	(5)	(6)
LTCI	0.243*** (0.083)	0.145* (0.087)	0.086* (0.048)	0.133*** (0.048)	−0.251*** (0.056)	−0.178*** (0.055)
*R*-squared	0.101	0.159	0.164	0.129	0.206	0.193
Control variables	Controlled	Controlled	Controlled	Controlled	Controlled	Controlled
Sample size	11,475	11,416	11,475	11,416	11,475	11,416

*p < 0.1 and ***p < 0.01. Standard errors clustered at the individual level are reported in parentheses.

### 5.5 Heterogeneity analysis

“Ensuring basic protection and broad coverage” is one of the key objectives of LTCI. However, due to differences in residence and individual characteristics, the association between LTCI policy exposure and women’s time allocation may vary across groups. To further examine heterogeneity, the sample is divided by residence, age, and income level, and the Model 2 specification is employed to estimate subgroup-specific associations between LTCI policy exposure and women’s time allocation.

#### 5.5.1 Residence

Table 9 reports the heterogeneous associations between LTCI policy exposure and time allocation across rural and urban women.

**Table 9:** Heterogeneous results of residence.

Group categories	Variable	Market labor time	Leisure time	Household labor time
		(1)	(2)	(3)
Rural	LTCI	−0.054 (0.181)	0.183* (0.110)	−0.145 (0.117)
	Sample size	5,708	5,708	5,708
Urban	LTCI	0.166* (0.098)	0.124** (0.054)	−0.188*** (0.063)
	Sample size	5,703	5,703	5,703

*p < 0.1, **p < 0.05, and ***p < 0.01. Standard errors clustered at the individual level are reported in parentheses.

The results show that, in terms of market labor time, LTCI policy exposure is significantly associated with increased market labor time among urban women, while the association among rural women is not statistically significant.

In terms of leisure time, LTCI policy exposure is significantly associated with increased leisure time among both rural and urban women. Based on the marginal effects calculated from Model 2, the estimated associations are 0.464 for rural women and 0.200 for urban women, respectively.

In terms of household labor time, LTCI policy exposure is significantly associated with reduced household labor time among urban women, while the association among rural women is not statistically significant.

#### 5.5.2 Age

We define women born after 1980 as the “new generation” and those born in 1980 or earlier as the “older generation” (37), and use subgroup regressions to examine age heterogeneity in the association between LTCI policy exposure and women’s time allocation. Table 10 reports the corresponding estimates.

**Table 10:** Heterogeneous results of age.

Group categories	Variable	Market labor time	Leisure time	Household labor time
		(1)	(2)	(3)
New generation	LTCI	0.000 (0.086)	0.152*** (0.051)	−0.170*** (0.058)
	Sample size	7,889	7,889	7,889
Old generation	LTCI	0.971*** (0.315)	0.058 (0.150)	−0.323* (0.181)
	Sample size	3,548	3,548	3,548

*p < 0.1 and ***p < 0.01. Standard errors clustered at the individual level are reported in parentheses.

In terms of market labor time, LTCI policy exposure is significantly associated with increased market labor time among older-generation women, while the association among new-generation women is not statistically significant.

In terms of leisure time, LTCI policy exposure is significantly associated with increased leisure time among new-generation women, while the association among older-generation women is not statistically significant.

In terms of household labor time, LTCI policy exposure is significantly associated with reduced household labor time among both new-generation and older-generation women. Based on the marginal effects calculated from Model 2, the estimated associations are −0.134 for new-generation women and −0.259 for older-generation women, respectively.

#### 5.5.3 Income level

We define women with a relative income level of average or above as the high-income group, and those below average as the low-income group, and use subgroup regressions to examine income heterogeneity in the association between LTCI policy exposure and women’s time allocation. Table 11 reports the corresponding estimates.

**Table 11:** Heterogeneous results of income level.

Group categories	Variable	Market labor time	Leisure time	Household labor time
		(1)	(2)	(3)
High-income	LTCI	0.123 (0.107)	0.105** (0.053)	−0.252*** (0.069)
	Sample size	4,839	4,839	4,839
Low-income	LTCI	0.303** (0.152)	0.267*** (0.092)	0.030 (0.092)
	Sample size	6,539	6,539	6,539

**p < 0.05 and ***p < 0.01. Standard errors clustered at the individual level are reported in parentheses.

In terms of market labor time, LTCI policy exposure is significantly associated with increased market labor time among low-income women, while the association among high-income women is not statistically significant.

In terms of leisure time, LTCI policy exposure is significantly associated with increased leisure time among both high-income and low-income women. Based on the marginal effects calculated from Model 2, the estimated associations are 0.341 for high-income women and 0.403 for low-income women, respectively.

In terms of household labor time, LTCI policy exposure is significantly associated with reduced household labor time among high-income women, while the association among low-income women is not statistically significant.

### 5.6 Potential mechanisms

Using the propensity score matched sample, this study examines the potential mechanisms through which LTCI affects women’s time allocation. The results are shown in Table 12.

**Table 12:** Potential mechanism analysis.

Variable	The substitution effect			The income effect	
	Care	Market labor time	Leisure time	Financial support	Household labor time
	(1)	(2)	(3)	(4)	(5)
LTCI	−0.070*** (0.022)	0.054 (0.098)	0.108* (0.056)	−0.347** (0.160)	−0.241*** (0.056)
Care		−0.202*** (0.077)	0.060 (0.049)		
Financial support					0.031*** (0.003)
*R*-squared	0.200	0.089	0.135	0.061	0.212
Control variables	Controlled	Controlled	Controlled	Controlled	Controlled
Sample size	3,296	3,296	3,296	11,416	11,416

*p < 0.1, **p < 0.05, and ***p < 0.01. Standard errors clustered at the individual level are reported in parentheses.

In terms of the substitution effect, Column (1) of Table 12 shows that LTCI significantly reduces women’s caregiving burden. Furthermore, the results in Column (2) show that caregiving burden is significantly negatively associated with market labor time, indicating that a reduction in caregiving burden may be associated with an increase in market labor time. However, Column (3) shows that the relationship between caregiving burden and leisure time is not statistically significant.

In terms of the income effect, Column (4) of Table 12 shows that LTCI significantly reduces women’s upward intergenerational financial support. Furthermore, the results in Column (5) show that upward intergenerational financial support is significantly positively associated with household labor time, suggesting that a decline in intergenerational financial burden may be associated with a reduction in household labor time.

Overall, LTCI may affect different dimensions of women’s time allocation through channels related to reductions in caregiving burden and upward intergenerational financial support.

### 5.7 Further analysis

Intergenerational emotional bonds are often reflected in face-to-face meetings and phone calls. Accordingly, this study measures intergenerational emotional bonds using intergenerational closeness, frequency of face-to-face meetings, and frequency of phone calls. Using the propensity score matched sample, this study further examines the effects of LTCI on these indicators.

As shown in Column (1) of Table 13, LTCI significantly increases intergenerational closeness. In terms of specific behaviors, the results in Columns (3) and (5) show that LTCI significantly increases the frequency of face-to-face meetings between women and their parents. Although the frequency of phone calls does not change significantly, there is no evidence of reduced communication. Overall, these results suggest that LTCI is associated with stronger intergenerational emotional bonds between women and their parents to some extent.

**Table 13:** Further analysis.

Variable	Intergenerational closeness		Frequency of face-to-face meetings		Frequency of phone calls	
	(1)	(2)	(3)	(4)	(5)	(6)
LTCI	0.290*** (0.080)	0.252*** (0.081)	0.901*** (0.118)	0.865*** (0.119)	0.103 (0.113)	0.064 (0.112)
Leisure time		0.285*** (0.029)		0.271*** (0.032)		0.288*** (0.029)
*R*-squared	0.098	0.119	0.079	0.093	0.201	0.217
Control variables	Controlled	Controlled	Controlled	Controlled	Controlled	Controlled
Sample size	11,416	11,416	11,416	11,416	11,416	11,416

***p < 0.01. Standard errors clustered at the individual level are reported in parentheses.

To further explore the possible role of leisure time, this study includes leisure time in the analysis. The results in Columns (2) and (4) of Table 13 show that LTCI is associated with an increase in women’s leisure time, and that leisure time is positively associated with intergenerational closeness and the frequency of face-to-face meetings.

Taken together, these results provide support for Hypothesis 3.

## 6 Discussion

Based on the above empirical results, we further discuss the following aspects.

Overall, long-term care insurance significantly affects the structure of women’s time allocation. In terms of the magnitude of change, adjustments in market labor time and household labor time are relatively pronounced. This pattern may reflect a shift in women’s time allocation from household labor to market labor after the implementation of the LTCI pilot program, while the change in leisure time is comparatively smaller. This finding is consistent with previous studies on LTCI and labor supply. Geyer and Korfhage (38) suggest that service benefits provided by LTCI help increase family caregivers’ market labor supply; Zhang et al. (19) further find that this positive effect is more pronounced among women. However, the allocation of leisure time and household labor time may be more closely related to individual preferences and household arrangements, and therefore needs to be interpreted in relation to group heterogeneity.

Heterogeneity analysis shows that the effects of LTCI on women’s time allocation vary across groups. In terms of market labor time, the effect is more pronounced among urban and low-income women, which is consistent with previous research (24). One possible explanation for the stronger effect among urban women is the difference in employment resources between urban and rural areas. According to the China Statistical Yearbook 2025, the urban–rural employment gap has continued to widen, with urban employment exceeding rural employment by about 175 million people in 2020 and about 213 million people in 2024. In this context, the limited capacity of rural labor markets to absorb workers may weaken the policy’s effect on labor supply to some extent. In addition, the stronger effect among low-income women may be related to their greater economic constraints. When the caregiving burden is alleviated, they may be more inclined to increase market labor time to improve their household economic situation.

In terms of leisure time, the effect of LTCI is more pronounced among rural women and new-generation women. From the residence dimension, one possible explanation is that employment opportunities are relatively limited in rural areas. After the family caregiving burden is reduced, the time freed up may not be fully converted into market labor time, but may instead be reflected more as an increase in leisure time. Feng (39), based on a study of northwestern Shanxi, China, also found that some rural women who accompanied their families to cities for their children’s schooling mainly spent their time in non-employment or leisure due to a lack of employment experience. From the age dimension, the more pronounced increase among new-generation women may be related to differences in time constraints across age groups. New-generation women are usually at a stage where marriage, childbearing, and career development overlap, with relatively strong time constraints; by contrast, some older-generation women may have already withdrawn from the labor market and have a higher stock of leisure time, making the marginal increase in leisure time smaller (40, 41).

In terms of household labor time, the effect of LTCI is more pronounced among high-income women and older-generation women. This finding is consistent with Bianchi et al. (42), who suggest that higher income levels are associated with higher opportunity costs of time and less time spent on household labor. In addition, Chen (43) shows that the number of children is significantly positively associated with women’s household labor time. Older-generation women may face heavier household labor burdens due to childrearing and intergenerational care responsibilities, which may help explain why the effect of LTCI on their household labor time is more pronounced.

Potential mechanism analysis shows that LTCI may increase women’s market labor time by reducing the family caregiving burden, which is consistent with previous research (19). However, the effect of this mechanism proxy in the leisure-time pathway is not significant, and future research may further verify this mechanism using more direct measures, such as formal care service utilization. In addition, LTCI may increase women’s disposable resources and household bargaining power by reducing their upward intergenerational financial support (28), thereby promoting the redistribution of household labor among family members (44). This explanation is consistent with the empirical results of this study.

Given the importance of intergenerational emotional bonds in the Chinese cultural context, this study further examines the relationship between LTCI and intergenerational emotional bonds. The results show that LTCI significantly increases intergenerational closeness and the frequency of face-to-face meetings between women and their parents. This finding can be understood in relation to changes in leisure time. Long-term caregiving not only consumes time, but may also increase caregiving pressure and weaken parent–child closeness (45). By providing care services, LTCI may free up some time for women with potential caregiving responsibilities. The increase in leisure time may also alleviate caregiving pressure (46, 47), thereby creating conditions for more positive intergenerational emotional interactions.

Based on the above discussion, this study proposes the following policy implications. First, in the context of the formal implementation of the long-term care insurance system, coverage equity and service accessibility should be improved. Particular attention should be paid to strengthening coordination between rural primary healthcare institutions and home-based care services. Second, benefit payment standards should be better coordinated across regions. These standards can be adjusted by considering regional income levels, the costs of formal care services, and differences in care resource supply. Third, coordination between long-term care insurance and employment support and income security policies should be strengthened. For rural women, reduced caregiving pressure does not necessarily translate into stable employment; therefore, employment services, skills training, and income support are still needed. Fourth, the professionalization of long-term care services should be promoted. High-intensity care should be gradually shifted to professional care workers, thereby reducing the family caregiving burden and creating more space for family members’ labor participation, rest and recovery, and emotional companionship.

This study has several limitations. First, due to differences in variable settings across CFPS waves, this study uses only the 2016 and 2018 data for analysis. Because data on intergenerational relationships are not available in 2014 and the 2020 questionnaire structure was substantially adjusted due to the COVID-19 pandemic, this study mainly identifies the short-term effects in the early stage of policy implementation and is unable to fully examine long-term effects.

Second, because data on actual participation in LTCI are not yet available, this study can only identify the average policy effect among the potentially affected population in pilot areas. It cannot distinguish actual beneficiaries from non-beneficiaries, which may lead to an underestimation of the actual policy effect.

Third, because information on parental disability is substantially missing in the CFPS questionnaire, this study does not further control for parental disability status or intergenerational co-residence. Therefore, the findings are more appropriately interpreted as the average effect of the pilot policy on women with potential caregiving responsibilities.

Fourth, this study measures leisure time only by summing a limited set of leisure activities, which makes it difficult to capture the structure and quality of leisure time. Therefore, the findings cannot fully indicate an improvement in leisure quality.

Fifth, the CFPS data lack information on formal care services, community service utilization, and the use of specific care subsidies, which limits the ability to directly test the mechanisms. Future research should further verify these mechanisms using more comprehensive data.

## 7 Conclusion

Based on data from the 2016 and 2018 waves of the China Family Panel Studies (CFPS), this study uses the long-term care insurance (LTCI) pilot program as a quasi-natural experiment to examine its effects on women’s time allocation and further analyze changes in intergenerational emotional bonds.

The results show that LTCI is significantly associated with changes in women’s time allocation, as reflected in increases in market labor time and leisure time, and a decrease in household labor time. These effects vary across groups defined by residence, age, and income. Potential mechanism analysis suggests that these changes may mainly result from LTCI reducing the family caregiving burden, thereby increasing women’s market labor time; meanwhile, LTCI may affect household labor time allocation by reducing upward intergenerational financial support. In addition, LTCI is associated with stronger intergenerational emotional bonds between women and their parents, with leisure time potentially playing a role.

The findings not only provide empirical evidence for the further improvement of the LTCI system, but also extend the understanding of its policy effects by focusing on women’s time allocation and family relationships.

## Data Availability

The original contributions presented in the study are included in the article/Supplementary material, further inquiries can be directed to the corresponding author.

## Footnotes

1 Including 15 pilot cities, namely Chengde (Hebei), Changchun (Jilin), Qiqihar (Heilongjiang), Shanghai, Nantong and Suzhou (Jiangsu), Ningbo (Zhejiang), Anqing (Anhui), Shangrao (Jiangxi), Qingdao (Shandong), Jingmen (Hubei), Guangzhou (Guangdong), Chongqing, Chengdu (Sichuan), and Shihezi (Xinjiang Production and Construction Corps), as well as two provinces, Shandong and Jilin.

2 Excluding samples from 10 provinces, namely Anhui, Sichuan, Hebei, Guangdong, Hubei, Jiangsu, Zhejiang, Jiangxi, Xinjiang, and Heilongjiang.

3 This study uses the 2016 and 2018 waves of the China Family Panel Studies (CFPS), with an initial sample of 71,626 observations. The sample selection proceeds as follows. First, 31,062 male observations are excluded. Second, 9,334 observations aged over 60 are excluded. Third, 11,003 observations with both parents deceased are excluded. Fourth, 76 observations with abnormal time-use values exceeding 168 h per week are excluded. Fifth, 2,211 observations with missing key variables are excluded. Sixth, 6,465 observations from provinces with only partial policy implementation are excluded in the policy identification stage. After these procedures, the final sample consists of 11,475 observations, including 1,426 in the treatment group and 10,049 in the control group.

## References

[1] Ministry of Civil Affairs and National Working Commission on Aging. Basic data bulletin of the fifth sample survey on the living conditions of older adults in urban and rural China. (2024). Available online at: https://wenku.baidu.com/view/2698e4a3f124ccbff121dd36a32d7375a417c6d7.html?_wkts_=1781677274634&needWelcomeRecommand=1 (Accessed June 17, 2026).

[2] Zhang Y Wang W. Forecast of the scale of disabled elderly population and their care time demand (in Chinese). Popul Res. (2021) 45:110–25.

[3] Ministry of Civil Affairs and National Working Commission on Aging. Basic data bulletin of the fifth sample survey on the living conditions of the elderly in urban and rural China. (2024). Available online at: http://www.crca.cn/images/20241017wdsjgb.pdf (Accessed March 27, 2026).

[4] Wu F. Main characteristics of family caregivers for the elderly in China and differences in care input: an analysis based on the third survey of women's social status in China (in Chinese). Collect Women's Stud. (2017) 2:5–13.

[5] Chen L Fan HL Zhao N Chu LL. Study on the impact of family elderly care on women's labor employment (in Chinese). Econ Res J. (2016) 51:176–89.

[6] Van Houtven CH Coe NB Skira MM. The effect of informal care on work and wages. J Health Econ. (2013) 32:240–52. doi: 10.1016/j.jhealeco.2012.10.006 23220459

[7] Rokicka M Zajkowska O. Informal elderly caregiving and time spent on leisure: evidence from time use survey. Ageing Int. (2020) 45:393–410. doi: 10.1007/s12126-020-09396-5

[8] Cheng M Yang H Yu Q. Impact of informal caregiving on caregivers’ subjective well-being in China: a longitudinal study. Arch Public Health. (2023) 81:209. doi: 10.1186/s13690-023-01220-1, 38057939 PMC10698909

[9] Liu L Dong X Zheng X. Parental care and married women's labor supply in urban China. Feminist Econ. (2010) 16:169–92. doi: 10.1080/13545701.2010.493717

[10] Hajek A König HH. Impact of informal caregiving on loneliness and satisfaction with leisure-time activities. Findings of a population-based longitudinal study in Germany. Aging Ment Health. (2019) 23:1539–45. doi: 10.1080/13607863.2018.1506739, 30328702

[11] China Consumers Association. Research report on the 2022 elderly care consumption survey project. (2022). Available online at: https://aimg8.dlssyht.cn/u/551001/ueditor/file/276/551001/1719449713475276.pdf (Accessed March 27, 2026).

[12] Huang Z Jian A Wang J Chen H. The effects of public long-term care insurance on the long-term care industry in China: a quasi-experimental study. BMC Geriatr. (2025) 25:276. doi: 10.1186/s12877-025-05933-6, 40281486 PMC12023454

[13] Kim HB Lim W. Long-term care insurance, informal care, and medical expenditures. J Public Econ. (2015) 125:128–42. doi: 10.1016/j.jpubeco.2014.12.004

[14] Lei X Bai C Hong J Liu H. Long-term care insurance and the well-being of older adults and their families: evidence from China. Soc Sci Med. (2022) 296:114745. doi: 10.1016/j.socscimed.2022.114745, 35093795

[15] Wang J Guan J Wang G. Impact of long-term care insurance on the health status of middle-aged and older adults. Health Econ. (2023) 32:558–73. doi: 10.1002/hec.4634, 36403228

[16] Mommaerts C. Long-term care insurance and the family. J Polit Econ. (2025) 133:1–52. doi: 10.1086/732887

[17] Long C Yang W Glaser K. Can long-term care insurance reduce catastrophic health and long-term care expenditures among older adults? A quasi-experimental study in China. Eur J Ageing. (2025) 22:25. doi: 10.1007/s10433-025-00861-1, 40461675 PMC12134244

[18] Coe NB Goda GS Van Houtven CH. Family spillovers and long-term care insurance. J Health Econ. (2023) 90:102781. doi: 10.1016/j.jhealeco.2023.102781, 37315472 PMC10533212

[19] Zhang L Dong J Li S Fang Y. The effect of long-term care insurance on labor force participation among informal caregivers: evidence from China. Humanit Soc Sci Commun. (2026) 13:339. doi: 10.1057/s41599-026-06658-6

[20] Qu Y Li Y Yin A Li J. Unlocking intergenerational support: the impact of long-term care insurance on elderly family dynamics. PLoS One. (2025) 20:e0337073. doi: 10.1371/journal.pone.0337073, 41270116 PMC12637977

[21] Wang X Tian W Zhan G He Y. The impact of long-term care insurance on intergenerational interaction behavior change in China. J Fam Econ Issues. (2025) 46:613–31. doi: 10.1007/s10834-024-09957-9

[22] Becker GS. A theory of the allocation of time. Econ J. (1965) 75:493–517. doi: 10.2307/2228949

[23] Ai JY Peng XZ. The impact of long-term care insurance on family members' labor supply (in Chinese). Insur Stud. (2024) 5:73–84. doi: 10.13497/j.cnki.is.2024.05.006

[24] Pei X Yang W Xu M. Examining the impact of long-term care insurance on the care burden and labor market participation of informal carers: a quasi-experimental study in China. J Gerontol B Psychol Sci Soc Sci. (2024) 79:gbae023. doi: 10.1093/geronb/gbae023, 38393966 PMC11227048

[25] Pollak RA. How bargaining in marriage drives marriage market equilibrium. J Labor Econ. (2019) 37:297–321. doi: 10.1086/698901

[26] Becker GS. A theory of social interactions. J Polit Econ. (1974) 82:1063–93. doi: 10.1086/260265

[27] Feng J Wang Z Yu Y. Does long-term care insurance reduce hospital utilization and medical expenditures? Evidence from China. Soc Sci Med. (2020) 258:113081. doi: 10.1016/j.socscimed.2020.113081, 32540515

[28] Wang L Liu J. The impact of long-term care insurance on family care for older adults: the mediating role of intergenerational financial support. PLoS One. (2024) 19:e0299974. doi: 10.1371/journal.pone.0312716 38781177 PMC11115205

[29] Zeng Y Brasher MS Gu D Vaupel JW. Older parents benefit more in health outcome from daughters’ than sons’ emotional care in China. J Aging Health. (2016) 28:1426–47. doi: 10.1177/0898264315620591, 26746225 PMC5947966

[30] Xu L Liu Y He H Fields NL Ivey DL Kan C. Caregiving intensity and caregiver burden among caregivers of people with dementia: the moderating roles of social support. Arch Gerontol Geriatr. (2021) 94:104334. doi: 10.1016/j.archger.2020.104334, 33516077

[31] Hebblethwaite S Norris J. Expressions of generativity through family leisure: experiences of grandparents and adult grandchildren. Fam Relat. (2011) 60:121–33. doi: 10.1111/j.1741-3729.2010.00637.x

[32] Hodge C Bocarro JN Henderson KA Zabriskie R Parcel TL Kanters MA. Family leisure: an integrative review of research from select journals. J Leis Res. (2015) 47:577–600. doi: 10.18666/jlr-2015-v47-i5-5705

[33] Kim EHW Lee C Do YK. The effect of adult children's working hours on visits to elderly parents: a natural experiment in Korea. Popul Res Policy Rev. (2019) 38:53–72. doi: 10.1007/s11113-018-9486-0

[34] Du F Zhao Y. Time use among urban women in China at different income levels. J Asian Econ. (2025) 96:101866. doi: 10.1016/j.asieco.2024.101866

[35] Jiang W Yang H. Health spillover studies of long-term care insurance in China: evidence from spousal caregivers from disabled families. Int J Equity Health. (2023) 22:191. doi: 10.1186/s12939-023-02001-6, 37723563 PMC10507894

[36] Zhang Y Cheng LG Liu ZB. Study on the impact of "new rural pension scheme" on the quality of elderly care for rural residents (in Chinese). China Econ Q. (2016) 15:817–44. doi: 10.13821/j.cnki.ceq.2016.01.17

[37] Li C. Children of the reform and opening-up: China’s new generation and new era of development. J Chin Sociol. (2020) 7:18. doi: 10.1186/s40711-020-00130-x

[38] Geyer J Korfhage T. Long-term care insurance and carers' labor supply–a structural model. Health Econ. (2015) 24:1178–91. doi: 10.1002/hec.3200, 26033403

[39] Feng X. Accompanying children to study: rural young women entering cities and the hidden expression of leisure life—an analysis based on the phenomenon of “entering cities to accompany children to study” in Xiaozhai township, northwestern Shanxi (in Chinese). China Youth Stud. (2017) 12:60–6. doi: 10.19633/j.cnki.11-2579/d.2017.0043

[40] Wang XB. Women's leisure: a new perspective on analyzing women (in Chinese). Zhejiang Acad J. (2002) 5:201–6. doi: 10.16235/j.cnki.33-1005/c.2002.05.031

[41] Xue B Fleischmann M Head J McMunn A Stafford M. Work-family conflict and work exit in later career stage. J Gerontol B. (2020) 75:716–27. doi: 10.1093/geronb/gby146, 30496506 PMC7768697

[42] Bianchi SM Sayer LC Milkie MA Robinson JP. Housework: who did, does or will do it, and how much does it matter? Soc Forces. (2012) 91:55–63. doi: 10.1093/sf/sos120, 25429165 PMC4242525

[43] Chen X. The gendered division of housework in China: parenthood effects and heterogeneity across parenthood stages. Popul Res Policy Rev. (2024) 43:30. doi: 10.1007/s11113-024-09872-9

[44] Doss C. Intrahousehold bargaining and resource allocation in developing countries. World Bank Res Obs. (2013) 28:52–78. doi: 10.1093/wbro/lkt001

[45] Lawrence RH Tennstedt SL Assmann SF. Quality of the caregiver–care recipient relationship: does it offset negative consequences of caregiving for family caregivers? Psychol Aging. (1998) 13:150–8. doi: 10.1037/0882-7974.13.1.150 9533197

[46] O’Shea E Timmons S O’Shea E Fox S Irving K. Respite in dementia: an evolutionary concept analysis. Dementia (London). (2019) 18:1446–65. doi: 10.1177/1471301217715325 28659025

[47] Conlin MM Caranasos GJ Davidson RA. Reduction of caregiver stress by respite care: a pilot study. South Med J. (1992) 85:1096–100. doi: 10.1097/00007611-199211000-00010, 1439947

