# Supplementary material for: Effects of long-term care insurance on women’s time allocation: evidence from pilot regions in China
Source: Front Public Health. 2026 Jul 20;14:1841498. doi: 10.3389/fpubh.2026.1841498 (PMC13429663; doi:10.3389/fpubh.2026.1841498)
Supplement: Supplementary file 1 [file Table_1.DOCX]

Supplementary Material

# Supplementary Figures and Tables

## Supplementary Tables

Table 1 Implementation details of pilot regions.

| **Region** | **Date** | **Policy-covered regions** | **Insured population** | **Benefit scope** |
| --- | --- | --- | --- | --- |
| Qingdao | 2012-7 | Urban | UEBMI and URBMI | Institutional care or home-based care expenses |
| Changchun | 2015-5 | Urban | UEBMI and URBMI | Institutional care expenses |
| Nantong | 2016-1 | All | UEBMI and URRBMI | Institutional care or home-based care expenses |
| Chengde | 2016-11 | Urban | UEBMI | Institutional care expenses |
| Jingmen | 2016-11 | All | UEBMI and URRBMI | Institutional care, hospital care, or home-based care expenses |
| Anqing | 2017-1 | Urban | UEBMI | Institutional care or home-based care expenses |
| Shanghai | 2017-1 | All | UEBMI and URRBMI | Institutional care, hospital care, community-based care, or home-based care expenses |
| Shangrao | 2017-1 | Urban | UEBMI | Institutional care, home-based care, or informal home care expenses |
| Shihezi | 2017-1 | All | UEBMI and URRBMI | Institutional care, home-based care, or informal home care expenses |
| Suzhou | 2017-6 | All | UEBMI and URRBMI | Institutional care, community-based care, or home-based care expenses |
| Chengdu | 2017-7 | Urban | UEBMI | Institutional care, home-based care, or informal home care expenses |
| Guangzhou | 2017-8 | Urban | UEBMI | Institutional care or home-based care expenses |
| Qiqihar | 2017-10 | Urban | UEBMI | Institutional care or home-based care expenses |
| Ningbo | 2017-12 | Urban | UEBMI | Institutional care or home-based care expenses |
| Chongqing | 2017-12 | Urban | UEBMI | Institutional care or home-based care expenses |

Notes: This table is compiled based on the implementation plans (or policy documents) of the long-term care insurance pilot programs in 15 pilot regions. Due to space limitations, the detailed municipal-level regulations of key provinces are not presented in this article.
